# Concentrations of Serum Per- and Polyfluoroalkyl Substances and Lipid Health in Adolescents: A Cross-Sectional Study from the Korean National Environmental Health Survey 2018–2020

**DOI:** 10.3390/toxics13020091

**Published:** 2025-01-25

**Authors:** Min-Won Shin, Habyeong Kang, Shin-Hye Kim

**Affiliations:** 1 Department of Pediatrics, Inje University Sanggye Paik Hospital, Seoul 01757, Republic of Korea; s5727@paik.ac.kr; 2 Department of Preventive Medicine, College of Medicine, Hanyang University, Seoul 04763, Republic of Korea; habyeongkang@gmail.com

**Keywords:** biomonitoring, lipid, endocrine disruptors, adolescents, perfluoroalkyl substances

## Abstract

Emerging evidence indicates that environmental exposure to per- and polyfluoroalkyl substances (PFASs) may influence lipid metabolism, though studies on adolescents remain scarce. This study aimed to investigate the association between PFAS mixture exposure and lipid profiles in Korean adolescents. Using data from the Korean National Environmental Health Survey (2018–2020), we analyzed 824 adolescents aged 12–17 years. Serum concentrations of PFAS, including perfluorooctanoic acid (PFOA), perfluorooctane sulfonic acid (PFOS), perfluorononanoic acid (PFNA), perfluorohexane sulfonic acid (PFHxS), and perfluorodecanoic acid (PFDeA), and lipid profiles were assessed. In multivariate regression models, PFDeA and PFNA were positively associated with elevated total cholesterol and low-density lipoprotein cholesterol levels, and PFDeA was associated with hypercholesterolemia risk in boys. In girls, PFDeA was associated with higher high-density lipoprotein cholesterol and lower triglycerides, though no significant association with hypercholesterolemia risk was observed. Bayesian kernel machine regression demonstrated positive associations between PFAS mixture exposure and hypercholesterolemia risk in boys but not in girls. The quantile g-computation model also demonstrated an odds ratio (OR) of 1.47 (95% CI: 0.99–2.19, *p* = 0.057) for PFAS mixture exposure in boys, suggesting borderline statistical significance. These findings suggest that PFAS exposure may disrupt lipid metabolism, elevating hypercholesterolemia risk in adolescents, particularly boys.

## 1. Introduction

Hypercholesterolemia, defined by high blood cholesterol levels, particularly low-density lipoprotein cholesterol (LDL-C), non-high-density lipoprotein cholesterol (non-HDL-C), and total cholesterol (TC), is a major risk factor for cardiovascular disease, including ischemic heart diseases and strokes [1]. The early hypercholesterolemia onset in adolescence raises significant concern, as lipid profiles established during this critical period often persist into adulthood, heightening the risk of early atherosclerotic changes and lifelong cardiovascular disease [1].

Recently, hypercholesterolemia prevalence among Korean adolescents has risen substantially, a trend largely attributed to the increasing adoption of Westernized dietary and lifestyle habits [2]. This shift, characterized by greater consumption of high-fat, high-sugar foods along with reduced physical activity, has contributed to growing rates of obesity and metabolic syndrome, both of which are strongly associated with dyslipidemia, including hypercholesterolemia [2]. Although lifestyle factors are central to hypercholesterolemia development, emerging evidence suggests that environmental exposures are significant yet under-recognized contributors to its increasing prevalence. In particular, growing evidence suggests that per- and polyfluoroalkyl substances (PFAS) exposure may negatively impact metabolic health, including lipid profile dysregulation.

PFAS, a group of artificial chemicals, are prevalent in industrial and consumer goods for their distinct qualities, such as their resistance to water, oil, and high temperatures [3]. Humans are frequently exposed to PFAS via polluted water and food sources, the use of consumer products, such as non-stick cookware, and direct contact with treated fabrics and packaging materials [3]. Studies have consistently detected PFAS in the blood samples of the general population, highlighting the pervasive nature of this exposure [4]. The potential adverse health outcomes of PFAS exposure include endocrine disruption, immune system impairment, and an elevated risk of certain cancers [4]. Of particular interest is the association between PFAS exposure and hypercholesterolemia, which may offer insights into how environmental pollutants contribute to metabolic disorders and cardiovascular risk.

Multiple studies have suggested the potential unfavorable impacts of PFAS exposure on human lipid health. A comprehensive meta-analysis of 29 studies consistently demonstrated positive associations between PFAS exposure and lipid levels in adults, with perfluorooctane sulfonate (PFOS) and perfluorooctanoic acid (PFOA) being the most extensively studied compounds. These studies reported significant associations between PFAS exposure and increased levels of TC, triglycerides (TGs), and LDL-C, although the magnitude of these increases was generally modest [5]. A recent contribution to this body of research reported that increased serum PFAS mixtures correlated with elevated trends in TC and LDL-C over a 15-year life span in a large cohort of mid-life United States (US) women [6]. This finding suggests that PFAS exposure may have long-term consequences on lipid health, potentially contributing to elevated cardiovascular disease risk later in life.

However, despite these findings, evidence regarding PFAS exposure’s impact on lipid health remains inconsistent, particularly in pediatric populations. Studies involving children and adolescents have reported mixed results, with some studies indicating a positive association between PFAS exposure and altered lipid profiles [7,8,9,10], while others found no significant associations [11,12]. Research on Norwegian adolescents found that higher serum PFOS, perfluorononanoate (PFNA), perfluorodecanoate (PFDeA), and perfluoroundecanoate (PFUnDA) levels correlated with increased apolipoprotein B, TC, and LDL-C [7]. Conversely, two cross-sectional studies—one involving girls exposed to PFAS through drinking water and another involving the US general adolescent population—reported null associations between most PFAS compounds and serum lipid profiles [11,12].

These inconsistencies may be attributed to various effect modifiers, including ethnicity/race and sex. Ethnicity/race has been proposed as a factor that modifies the health effects of PFAS exposure through genetic, dietary, and environmental differences. For example, differences in the consumption of diet across ethnic groups can influence the types and levels of PFAS exposure, with certain populations being more exposed to specific PFAS compounds. In addition, genetic susceptibility to the same PFAS exposure levels can differ between ethnic and racial groups, further contributing to variations in health outcomes. The Multiethnic Cohort Study highlighted the significance of assessing PFAS health effects in racially and ethnically diverse populations, discovering evidence of increased kidney cancer risk among African American participants with higher PFAS levels compared with those of other ethnicities [13]. The potential effects of PFAS exposure on lipid metabolism in the Asian pediatric population remain largely unexplored, presenting a significant gap in the literature. Given the potential distinctions in physiology and diet, particularly the high seafood consumption in Asian populations, further research is needed to understand how PFAS exposure may influence lipid metabolism in this understudied group.

Sex-related differences in lipid profiles may also influence the association between PFAS exposure and dyslipidemia. Female adolescents typically exhibit higher TC, LDL-C, and HDL-C levels than their male counterparts do [14], whereas they tend to have lower serum PFAS levels owing to menstruation [15]. These biological differences suggest that sex could be an important modifier of the PFAS-related effects on lipid regulation; however, a few studies have specifically investigated this potential interaction.

Furthermore, although PFAS compounds may exert additive or synergistic effects when present as mixtures, most studies have primarily focused on the effects of individual PFAS compounds on lipid health. Specifically, a lack of comprehensive analysis on the joint effects of multiple PFAS exposures exists, including potential interactions between different PFAS compounds [16]. This gap is particularly evident in pediatric populations, where such investigations remain scarce.

Given the inconsistent findings and the potential influence of various effect modifiers, more comprehensive studies are needed to elucidate the relationship between PFAS exposure and lipid health in adolescents. The Korean National Environmental Health Survey (KoNEHS) 2018–2020 provides an opportunity to investigate these associations in a representative sample of South Korean adolescents. This study aimed to explore the associations between serum PFAS concentrations and lipid profiles in adolescents, considering potential effect modifiers such as sex differences and the joint effects of multiple PFAS compounds.

## 2. Materials and Methods

### 2.1. Study Population

Our study utilized data obtained from the fourth cycle of the KoNEHS (2018–2020), an extensive nationwide biomonitoring investigation designed to systematically monitor and evaluate environmental chemical exposures among the Korean general population. It employed a stratified, multistage probability sampling method ensuring comprehensive demographic representation across South Korea [17].

The survey collected a comprehensive range of data encompassing factors such as socioeconomic status and health conditions, obtained through interviews based on questionnaires that specifically investigated environmental chemical exposure. Furthermore, the survey comprised physical assessments to acquire anthropometric measurements and laboratory examinations to substantiate the information collected. This study utilized information from the fourth cycle of the KoNEHS since it was the sole cycle that measured serum PFAS levels. Initially, a pediatric population of adolescents aged 12–17 years, totaling 828 participants, was included in KoNEHS 2018–2020. After applying exclusion criteria, three participants who did not undergo blood sampling or PFAS concentration testing and one participant with severely elevated serum TG levels (≥800 mg/dL), whose LDL-C could not be accurately calculated using the Martin–Hopkins formula, were excluded. This resulted in a final dataset of 824 participants. No participant reported lipid-lowering medication use or had a known history of endocrine disorders that affect lipid metabolism, such as thyroid dysfunction or diabetes mellitus.

The KoNEHS adhered to the ethical principles of the Declaration of Helsinki. Written consent was collected from all participants and their legal guardians before participation. The study protocol received approval from the Institutional Review Board of Inje University Sanggye Paik Hospital (IRB No. 2024-02-005).

### 2.2. Determination of Serum Lipid Profiles

Participant blood samples were drawn using venipuncture and stored in serum separator tubes. Fasting was not mandated prior to blood sample collection. Samples were left to clot at ambient temperature for 30 min, followed by centrifugation at 3000 rpm for 10 min. The separated serum was used to determine lipid profiles using an ADVIA 1800 Clinical Chemistry System (Siemens Medical Sol., Malvern, PA, USA). The ADVIA 1800 system is an automated analyzer designed for high-throughput biochemical assays, including lipid profiling. A colorimetric enzymatic assay was used to measure TC, beginning with cholesterol ester conversion to free cholesterol, which is then oxidized to produce a color intensity directly proportional to cholesterol concentration, with absorbance read at 505/694 nm. For HDL-C measurement, LDL-C and very low-density lipoprotein (VLDL) were selectively precipitated, with the remaining HDL-C measured by a similar colorimetric enzymatic assay. TGs were measured by hydrolyzing them to glycerol and free fatty acids, with the ensuing reactions forming a colored complex, whose absorbance at 505/694 nm indicates the TG concentration. To obtain non-HDL-C, the HDL-C value was subtracted from the TC as follows: non-HDL-C = TC − HDL-C [18]. LDL-C was calculated using an extended Martin/Hopkins equation, which applies a strata-specific median ratio of TG to VLDL-C. This method is based on 40 TG categories and 6 non-HDL-C categories, resulting in a 240-cell stratification, as detailed in a previous study [19]. This approach enhances the accuracy of LDL-C estimation, particularly in individuals with elevated TG levels and in non-fasting states.

Subnormal lipid profiles were defined according to the cut-off values established by the 2017 clinical practice guidelines for dyslipidemia in Korean children and adolescents as follows: TC ≥ 170 mg/dL, LDL-C ≥ 110 mg/dL, and non-HDL-C ≥ 120 mg/dL [1]. Hypercholesterolemia was defined as meeting one or more of these subnormal lipid profile criteria.

### 2.3. Measurement of Serum PFAS Concentrations

The serum samples were processed by aliquoting into polypropylene tubes and stored at −80 °C until analysis. For PFAS quantification, specifically targeting compounds such as PFOA, PFOS, perfluorohexane sulfonic acid (PFHxS), PFNA, and PFDeA, high-performance liquid chromatography-tandem mass spectrometry (HPLC-MS/MS) was utilized (PerkinElmer, Waltham, MA, USA). Sample preparation involved adding internal standards along with 800 µL of acetonitrile to each serum sample, followed by a 10 s agitation to facilitate protein precipitation. The samples were then centrifuged, after which 800 µL of the supernatant was collected and further concentrated under nitrogen gas. The final concentrated samples were diluted in a 50% methanol solution, preparing them for HPLC-MS/MS analysis.

To enhance the methodological rigor and ensure the validity of the analytical procedures, quality control (QC) measures were systematically implemented. The QC protocol incorporated the use of certified reference materials such as PFHxS, PFOA, PFNA, PFOS, and PFDeA (Sigma-Aldrich, Saint Louis, Missouri, USA) to establish calibration curves and assess the quantification process accuracy. To further refine analysis precision, isotopically labeled internal standards, specifically PFOA-^13^C_8_ and PFOS-^13^C_8_, were obtained from Cambridge Isotope Laboratories and integrated into the sample preparation workflow. In addition to the internal quality assurance measures, the performance of the method was subjected to external validation through participation in the German External Quality Assessment Scheme. This interlaboratory comparison program provided an independent assessment of the accuracy of the method and comparability with other laboratories. The detection limits for PFOS, PFOA, PFHxS, PFNA, and PFDeA were 0.056, 0.050, 0.071, 0.019, and 0.017 µg/L, respectively. For concentrations below the detection limit, the square root of the limit was used for imputation [20].

### 2.4. Covariates

Data were collected using standardized survey methodologies to assess sociodemographic variables, including sex, age, and family income, stratified into the following three categories: <3, 3–5, and >5 million KRW/month. The survey also examined various lifestyle aspects. Alcohol intake was classified based on frequency, distinguishing between individuals who consumed less than one drink per week and those who had one or more drinks weekly. Smoking status was categorized as never smoked, former smoker, or current smoker. Physical activity was assessed through the walking routines of participants, with a threshold of <150 min per week to ≥150 min. In addition, the frequency of mass-produced food consumption was assessed in the following four categories: <twice, two to three times, four to six times, and ≥seven times per week. Quartile analysis was used to determine seafood consumption frequency. The intake of mass-produced food items such as instant noodles, hamburgers, frozen foods, popcorn, and canned goods served as a proxy for high-calorie consumption.

Anthropometric assessments were performed with participants wearing light attire and without footwear. Heights and weights were recorded to the nearest 0.1 cm and kg, respectively, using a stadiometer and a calibrated digital scale. Waist circumference (WC) was assessed at the midpoint between the lower rib and the top of the iliac crest using a nonelastic tape measure. Body mass index (BMI) was calculated as weight in kilograms divided by the square of height in meters. Obesity status was classified according to the 2017 Korean National Growth Charts for children and adolescents, using the following sex- and age-specific BMI percentile values: normal weight (<85th percentile) and overweight and obese (≥85th percentile) [21]. BMI z-scores were derived using the Lambda-Mu-Sigma method. Central obesity was defined based on sex- and age-specific WC percentiles, with a cut-off at ≥90th percentile [22]. The final dataset was complete with no missing covariates among participants, eliminating the need for additional preprocessing steps to handle missing data.

### 2.5. Statistical Analysis

Statistical analyses were performed using R software (version 4.3.3). Results were considered statistically significant if the two-sided *p*-value was <0.05. The study design adhered to the KoNEHS guidelines, incorporating complex survey elements such as strata, primary sampling units, and sampling weights. Due to the right-skewed non-normal distribution of PFAS concentrations, a logarithmic transformation was applied before analysis. Spearman’s correlation coefficients (ρ) were calculated to evaluate the associations between serum PFAS concentrations. PFAS levels were categorized into three groups based on tertiles, using the lowest tertile as the baseline reference for comparison. TG also underwent log transformation to approximate a normalized distribution, whereas the other lipid parameters exhibited normal distribution and, consequently, did not require transformation.

Sex-stratified analyses were conducted to account for lipid profile differences between male and female adolescents. Multivariate linear regression assessed the association between serum PFAS levels and lipid profiles, while multivariate logistic regression evaluated the associations between PFAS concentrations and the likelihood of subnormal lipid profiles. Each model was adjusted for potentially confounding variables, including age, central obesity, family income, smoking, alcohol habits, walking habits, consumption of mass-produced food items/seafood, and menstruation years for girls.

To assess the impact of multiple PFAS exposures on hypercholesterolemia, Bayesian kernel machine regression (BKMR) and quantile g-computation models were used. BKMR was chosen for its strength in modeling complex interactions and non-linear relationships between exposures, allowing for a nuanced evaluation of environmental health effects [23]. The posterior inclusion probability (PIP) in BKMR quantifies the relevance of each exposure in the model, with higher PIP values indicating a stronger influence on the outcome. BKMR result interpretation typically involves comparing the exposure–response relationships when all PFAS levels are set to a specific percentile against when they are set to the 50th percentile. In this analysis, PFAS concentrations underwent log transformation and standardization. BKMR was then applied, utilizing 50,000 Monte Carlo iterations across a 260-knot grid, to comprehensively examine the exposure–response relationship.

Quantile g-computation offers a robust alternative to BKMR by directly assessing the effects of a quantile increase in chemical mixtures on health outcomes. Unlike BKMR, which models non-linear relationships and interactions, quantile g-computation provides a clear interpretation of how each exposure level increment affects the outcomes, enhancing a better understanding of exposure–response dynamics. The contribution of each PFAS compound to the overall mixture effect was quantified by weights, with the sum of weights normalized to one [24]. Positive weights imply that a chemical exposure increase is associated with an increase in health outcomes, while negative weights suggest an inverse relationship, where higher exposure correlates with a decrease in the outcome. In this study, a binomial distribution was used as the link function, and tertiles were chosen for the quantile division owing to the absence of significant non-linear patterns in most dose–response relationships. Two hundred bootstrap iterations were conducted to enhance the robustness of the model. The results are interpreted as the combined effect of a one-tertile increase in the PFAS mixture on hypercholesterolemia outcomes. Owing to the limitations of both BKMR and quantile g-computation methodologies, survey-weighted analyses were not included. BKMR and quantile g-computation methods were conducted using the ‘bkmr’ (version 0.2.2) and ‘qgcomp’ packages (version 2.15.2) in R.

## 3. Results

### 3.1. Participant Characteristics and Lipid Profiles

Table 1 summarizes the demographic and biochemical characteristics of 824 adolescent participants (383 boys and 441 girls) with a mean age of 14.5 years (±0.2 standard error [SE]). Central obesity was observed in 15.8% of the participants. The following sex differences were observed in lifestyle behaviors: boys had a higher smoking prevalence (16.4% vs. 6.1%) and greater mass-produced food consumption (33.1% vs. 18.7%) than girls did. Lipid profile assessments revealed considerable subnormal lipid profile status within the study population as follows: 27.3% had elevated TC (TC ≥ 170 mg/dL), 8.7% had high LDL-C (LDL-C ≥ 110 mg/dL), and 22.3% had elevated non-HDL-C (non-HDL-C ≥ 120 mg/dL). Notably, 29.8% of the population was identified as having hypercholesterolemia, with a significant female predominance (35.4% vs. 24.4%).

### 3.2. Distribution of Serum PFAS Concentrations

Table S1 displays the distribution of serum concentrations for five PFAS compounds, each detected at 100% in the study population. Geometric mean concentrations (µg/L) were observed as follows: PFOS exhibited the highest concentration at 7.97 µg/L, followed by PFOA at 3.66 µg/L, PFHxS at 2.52 µg/L, PFNA at 0.92 µg/L, and PFDeA at 0.45 µg/L. The specific cut-off values for PFAS tertiles are provided in Table S2. Notably, serum PFAS concentrations were significantly higher in boys than in girls, as detailed in Table S2. Furthermore, our correlation analysis presented in Figure S1 revealed strong associations between the serum concentrations of these compounds, particularly between PFNA and PFDeA, where the correlation coefficient was notably high (ρ = 0.84).

### 3.3. Association Between Individual Serum PFAS Concentrations and Lipid Profiles

**Figure 1:**
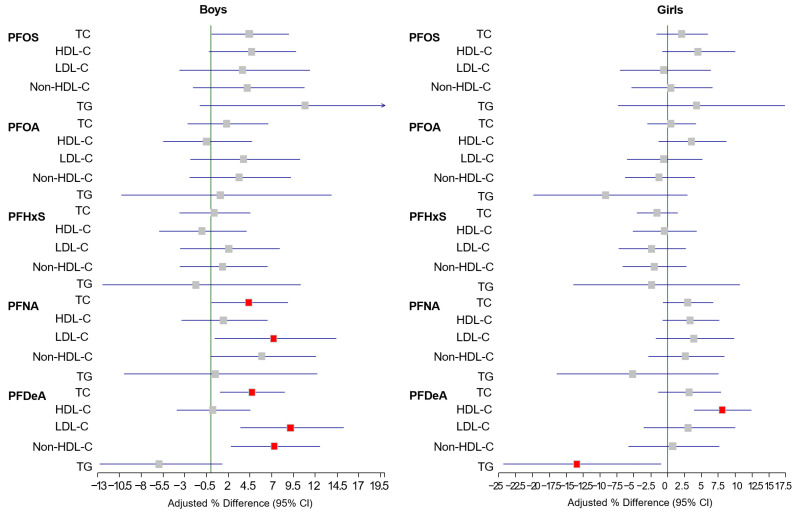
Adjusted percentage differences in lipid profiles of the highest tertiles of per- and polyfluoroalkyl substance (PFAS) concentrations compared with the lowest tertiles. Adjusted for age, central obesity, family income, smoking status, alcohol habits, walking habits, consumption of mass-produced food items/seafood, and menstruation years (girls). Squares represent adjusted percentage differences, and horizontal lines represent 95% confidence intervals. Red squares represent statistically significant differences (*p* < 0.05). CI, confidence interval; PFOA, perfluorooctanoic acid; PFOS, perfluorooctane sulfonic acid; PFNA, perfluorononanoic acid; PFHxS, perfluorohexane sulfonic acid; PFDeA, perfluorodecanoic acid; TC, total cholesterol; LDL-C, low-density lipoprotein cholesterol; HDL-C, high-density lipoprotein cholesterol; non-HDL-C, non-high-density lipoprotein cholesterol; TG, triglycerides.

Figure 1 presents the adjusted percentage changes in lipid profiles across different tertile groups of PFAS exposure among the study participants. In boys, the highest tertile of PFNA exposure was associated with significantly elevated TC and low-LDL-C levels compared with the lowest tertile. Similarly, those in the highest tertile of PFDeA exposure exhibited increased TC, LDL-C, and non-HDL-C levels than their counterparts did in the lowest tertile. Although the statistical significance was borderline, the highest PFOS quartile also showed a positive association with TC levels in boys. In contrast, among girls, the highest PFDeA tertile was linked to elevated HDL-C and reduced TG levels compared to the lowest tertile. Other PFNA concentrations did not demonstrate significant associations with lipid profiles in either sex.

**Figure 2:**
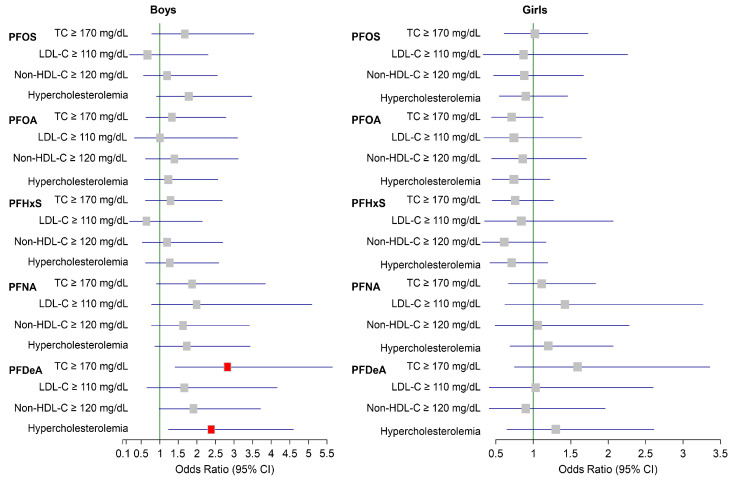
Odds ratios (95% CI) of subnormal lipid profiles of the highest tertiles of per- and polyfluoroalkyl substance (PFAS) concentrations compared with the lowest tertiles. Adjusted for age, central obesity, family income, smoking status, alcohol habits, walking habits, consumption of mass-produced food items/seafood, and menstruation years (girls). Squares represent adjusted odds ratios, and horizontal lines represent 95% confidence intervals. Red squares represent statistically significant differences (*p* < 0.05). CI, confidence interval; PFOA, perfluorooctanoic acid; PFOS, perfluorooctane sulfonic acid; PFNA, perfluorononanoic acid; PFHxS, perfluorohexane sulfonic acid; PFDeA, perfluorodecanoic acid; TC, total cholesterol; LDL-C, low-density lipoprotein cholesterol; non-HDL-C, non-high-density lipoprotein cholesterol.

Figure 2 shows the relationship between serum PFAS concentrations and the likelihood of subnormal lipid profiles, with adjustments made for relevant covariates. In boys, those in the highest tertile group of PFDeA showed significantly increased odds ratios (ORs), having TC levels ≥ 170 mg/dL (OR 2.82, 95% confidence interval [CI]: 1.41–5.64) and hypercholesterolemia (OR 2.38, 95% CI: 1.23–4.59) compared with those in the lowest group. However, no significant associations between any PFAS compound and subnormal lipid profiles were observed in girls.

### 3.4. Associations of PFAS Mixtures with Hypercholesterolemia: BKMR Model

Table S3 presents the PIPs for serum PFAS compounds in BKMR models by sex, evaluating hypercholesterolemia risk. In boys and girls, PFDeA exhibited the highest PIPs, highlighting the significant role of the compound in hypercholesterolemia risk. PFOA (PIP: 0.5234) and PFHxS (PIP: 0.4512) displayed relatively higher contributions in girls than in boys.

Figure S2 shows the sex-specific dose–response relationships between individual PFAS compounds and hypercholesterolemia risk in the BKMR model, with other PFAS compounds held at median levels. In boys, PFDeA displayed a strong positive association with increased hypercholesterolemia risk, while PFOS showed a modest positive association. Other compounds, including PFOA, PFHxS, and PFNA, exhibited weak or minimal negative associations with hypercholesterolemia risk. In girls, both PFDeA and PFNA showed positive associations with hypercholesterolemia. Conversely, PFHxS and PFOA were negatively associated with hypercholesterolemia risk.

**Figure 3:**
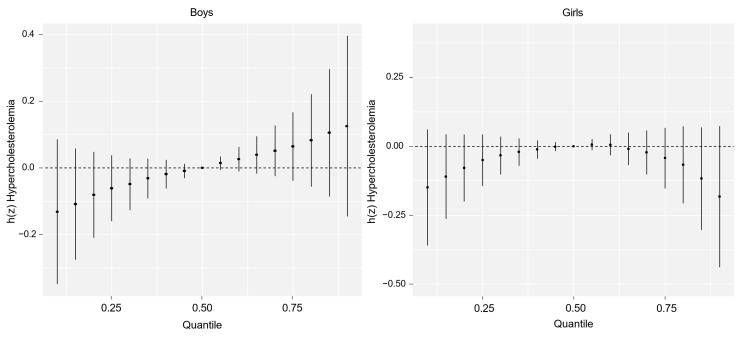
The combined effects of exposure to per- and polyfluoroalkyl substance (PFAS) mixtures on hypercholesterolemia were analyzed using the Bayesian kernel machine regression (BKMR) model. Each quantile level was compared with the 0.5th quantile as the baseline. The chart shows the estimated effects, along with the 95% confidence intervals. Adjusted for age, central obesity, family income, smoking status, alcohol habits, walking habits, consumption of mass-produced food items/seafood, and menstruation years (girls).

To better address the overall association of PFAS mixtures, Figure 3 highlights the joint association of PFAS mixture exposure with hypercholesterolemia risk in boys and girls using the BKMR model. In boys, a positive dose–response relationship was observed, with increasing PFAS mixture exposure associated with elevated hypercholesterolemia risk, albeit with the wide 95% CI variability. In contrast, the dose–response curve for girls remained relatively stable across all quantiles, indicating no significant combined association of PFAS mixture with hypercholesterolemia risk at varying PFAS exposure levels. This finding underscores sex-specific differences in the overall associations of PFAS mixtures.

### 3.5. Associations of PFAS Mixtures with Hypercholesterolemia: Quantile g-Computation Model

Figure S3 illustrates the relative weights of individual PFAS compounds in the PFAS mixture on hypercholesterolemia risk, as assessed by quantile g-computation models. In boys, PFDeA contributes the most to the positive association with hypercholesterolemia risk, followed by PFOS and PFHxS, while PFOA and PFNA showed a negative association. In contrast, for girls, PFDeA and PFNA exhibited the highest positive weights; however, PFOA and PFHxS showed negative weights.

Table 2 summarizes the joint associations of PFAS mixtures on hypercholesterolemia risk. In boys, the OR for PFAS mixture exposure was 1.472 (95% CI: 0.988–2.191; *p* = 0.057), indicating a borderline significant association. In girls, the OR was 1.003 (95% CI: 0.734–1.371; *p* = 0.985), showing no association. These findings suggest the stronger association of PFAS mixtures with hypercholesterolemia risk in boys.

## 4. Discussion

This study highlights significant sex-specific differences in the associations between PFAS exposure and hypercholesterolemia in Korean adolescents. In boys, PFDeA and PFNA showed strong positive associations with elevated TC and LDL-C, with PFDeA notably increasing hypercholesterolemia risk (OR 2.38, 95% CI: 1.23–4.59). In contrast, girls demonstrated a positive association between PFDeA and HDL-C and lower TG levels; however, no significant association was found between PFAS exposure and hypercholesterolemia risk. These findings suggest a clear sex difference in PFAS-related lipid dysregulation, which is more pronounced in boys, while the direction and magnitude of PFAS effects appear to differ between the sexes. Advanced mixture models, including BKMR and quantile g-computation, revealed positive associations between PFAS mixture exposure and hypercholesterolemia risk in boys, although these associations were less pronounced compared to the individual impact of PFDeA. This suggests that the combined effects of PFAS mixtures on lipid metabolism may be moderated by interactions between PFAS compounds, leading to non-linear or antagonistic effects.

Our study contributes to the growing body of research on the relationship between PFAS exposure and lipid metabolism, specifically focusing on the adolescent population. Although most existing studies focused on adult populations, several studies have consistently demonstrated the association between PFAS exposure and elevated cholesterol levels. For instance, Liu et al. conducted a meta-analysis revealing significant associations between PFOA and PFOS exposure and increased TC and LDL-C levels in adults [5]. This is in line with the findings from Canova et al., who observed that PFAS, particularly PFOS and PFOA, were linked to altered lipid parameters such as TC, HDL-C, and LDL-C, emphasizing the potential role of these substances in lipid dysregulation [9].

In adolescent-focused studies, the first large-scale studies by Frisbee et al. (2010) and Geiger et al. (2014) identified associations between PFOA, PFOS, and elevated TC and LDL-C among US pediatric populations, showing similar patterns to those in adults [10,25]. More recent studies have expanded on these observations by including other PFAS compounds and reporting potential associations with elevated lipid profiles. For example, Koshy et al. expanded on this by including other PFAS compounds, including PFHxS, PFNA, PFDeA, and PFUnDA, further supporting the potential lipid-disrupting effects in US children [26]. Similarly, Averina et al. reported that PFOS, PFNA, PFDeA, and PFUnDA concentrations were positively associated with not only TC and LDL-C but also with apolipoprotein B levels in Norwegian adolescents, with PFNA and PFDeA particularly linked to higher dyslipidemia risk [7]. Canova et al. (2021) added further evidence by evaluating highly exposed adolescents from Italy [16]. Their results revealed significant associations between PFOS, PFNA, and higher TC and LDL-C levels, similar to those found in studies from the US and Norway. These findings align with those of our study, where PFNA and PFDeA were associated with elevated TC and LDL-C levels in adolescents, highlighting the consistent role of specific PFAS in lipid dysregulation across diverse populations.

PFAS are known for their persistent bioaccumulative nature, and their impact on cholesterol metabolism is an emerging concern. PFAS can interfere with lipid metabolism through various pathways, particularly by interacting with nuclear receptors such as peroxisome proliferator-activated receptor (PPAR)-α [27], constitutive androstane receptor (CAR) [28], and pregnane X receptor (PXR) [27,29], which regulate lipid homeostasis. The stimulation of these nuclear receptors due to PFAS exposure may lead to elevated serum cholesterol levels through alterations in lipid production, bile acid metabolism, and cholesterol excretion [30,31]. In addition, emerging evidence suggests that PFAS compounds may induce oxidative stress and inflammation, further contributing to dyslipidemia [32]. These mechanisms are particularly relevant in adolescence, a period of rapid hormonal and metabolic changes that may amplify the effects of environmental pollutants on lipid health.

In this study, PFOS and PFOA—two of the most well-documented PFAS compounds—were not significantly associated with hypercholesterolemia risk in the Korean adolescent population. Notably, serum PFOS levels showed a borderline significant positive association with TC concentrations in boys, although no association was observed with LDL-C. This finding aligns with, but is weaker than, associations reported in adult populations worldwide. For example, the meta-analysis by Liu et al. found positive associations between PFOS and PFOA exposure and elevated cholesterol levels in adults [5]. A possible explanation for the weaker association in adolescents is that the lipid-disrupting effects of PFOS and PFOA may require prolonged, cumulative exposure, which may not yet be reflected in younger individuals. Moreover, adolescents undergo significant hormonal and metabolic changes that could modify their susceptibility to PFAS-induced lipid disturbances. Furthermore, global reductions in PFOS and PFOA exposure following the restrictions introduced by the Stockholm Convention could have influenced these findings [33]. Previous National Health and Nutrition Examination Survey studies, such as those by Geiger et al. [10] and Dong et al. [12], reported much higher serum PFOS and PFOA concentrations—up to 100% greater than those observed in our study—accompanied by stronger associations with increased cholesterol levels. In Korea, declining PFOS levels have been reported between 2006 and 2015, suggesting that reduced exposure could potentially exacerbate the sensitivity of the population to its lipid-altering effects over time [34]. Recent studies in adolescent populations from Norway [7] and Italy [16], where PFOS concentrations were comparable to or even lower than those found in our study, still demonstrated positive associations between PFOS exposure and both TC and LDL-C levels. This suggests that other factors, such as genetic predispositions, dietary habits, or environmental influences, play a significant role in modifying the health effects of PFAS exposure in adolescents of diverse ethnic backgrounds. These findings highlight the importance of future research to unravel the complex interplay between these variables and to better understand the population-specific effects of PFAS exposure.

In this study, significant positive associations between PFDeA and PFNA exposure and hypercholesterolemia in Korean adolescents were identified. The health effects of PFNA and PFDeA are less explored compared with PFOS and PFOA, primarily due to their historically lower concentrations in most populations. However, in this study, PFNA and PFDeA concentrations were notably higher than internationally reported levels [7,11,16,26], highlighting the importance of regional exposure patterns in assessing health risks. Positive associations of PFDeA and PFNA with serum lipid profiles were also reported in large-scale cross-sectional studies from Norway and Italy and longitudinal studies of mother-child cohorts from the Faroe Islands and the US [7,11,16,26]. These associations may result from the distinct bioaccumulation patterns of these longer-chain PFAS compounds and their differential activation of nuclear receptors, including PPARα, PXR, and CAR. PFNA and PFDeA tend to accumulate more effectively in the liver, where they exert hepatotoxic effects that impair the ability of the organ to regulate cholesterol synthesis, transport, and excretion. This disruption may promote lipid accumulation and increase serum cholesterol levels. A key aspect underlying these associations involves the differential activation of nuclear receptors by PFAS compounds. PPARα, PXR, and CAR are central to lipid metabolism and homeostasis, and each receptor responds differently to PFAS exposure [35]. PPARα enhances fatty acid β-oxidation, promoting energy metabolism in the liver; however, this increased lipid breakdown may trigger compensatory cholesterol synthesis as part of hepatic homeostasis [36]. Meanwhile, PXR, the primary regulator of drug metabolism, upregulates sterol regulatory element-binding protein 2, contributing to elevated LDL-C levels [37]. In contrast, CAR activation promotes cholesterol clearance by enhancing bile acid synthesis and stimulating excretion through bile, mitigating hypercholesterolemia [38]. Experimental studies reveal that PFAS compounds differ in their potential to activate PPARα [39] and bind to PXR [40]. Long-chain PFAS, PFNA, and PFDeA show stronger activation of these receptors than their shorter-chain counterparts, even though CAR activation remains consistently high across most PFAS compounds [28,41].

Using two different mixture models, PFDeA and PFNA were identified as the primary contributors to hypercholesterolemia risk in Korean adolescents, while the effects of PFAS mixtures showed only marginal statistical significance. This discrepancy may be attributed to the complex interactions among PFAS compounds in mixtures, which could potentially attenuate the robust effects observed in individual compounds. Emerging evidence suggests that PFAS mixtures behave differently from individual compounds, with nuclear receptor activation profiles shifting based on the composition and ratios of the PFAS involved. These interactions may produce non-linear outcomes—ranging from additive and synergistic to antagonistic effects—depending on the specific mixture [39]. For example, research on human liver cell models has shown that perfluorinated carboxylic acids act as full agonists of PPARα, while perfluorinated sulfonic acids act as partial agonists. Mixtures of these compounds displayed non-additive effects, with antagonistic interactions becoming evident at higher concentrations [27,42]. Similarly, studies in larval fish noted that PFOA could reduce the oxidative stress induced by PFOS, suggesting that certain PFAS may mitigate the toxic effects of others within mixtures [43]. Such interactions may explain the weaker combined effects observed in this study. Our findings from mixture models provide valuable insights into real-world exposure scenarios where individuals are exposed to multiple PFAS compounds simultaneously. These findings highlight the importance of advanced statistical approaches, such as BKMR and quantile g-computation, to account for the complex, non-linear relationships among PFAS compounds. However, the marginal significance observed in some mixture models emphasizes the need for further validation using larger and more diverse cohorts. Replication studies are crucial to confirm these associations and provide greater clarity on the interplay between individual PFAS compounds, mixtures, and hypercholesterolemia risk.

A key finding of this study was the sex-specific differences in how PFAS compounds impacted lipid profiles, with boys exhibiting stronger associations between PFAS exposure and lipid disturbances than girls. These differences may be partially attributed to hormonal variations, particularly estradiol’s role. Estradiol plays a crucial role in cholesterol metabolism by enhancing LDL-C clearance and promoting HDL-C production, which could offer protective effects in females [44]. Additionally, estradiol has been shown to modulate nuclear receptor activity, including PPAR-α, which regulates lipid metabolism. Evidence suggests that estradiol can inhibit PPAR-α-mediated transcriptional activity by interfering with coactivator recruitment [45], potentially reducing susceptibility to PFAS-induced lipid disturbances in females. This hormonal modulation may explain why males are more vulnerable to PFAS-related lipid dysregulation. Beyond estradiol’s effects, other mechanisms likely contribute to these sex-specific differences. Differential activation of nuclear receptors such as PXR and CAR by PFAS compounds may play a role. These receptors are involved in xenobiotic metabolism and lipid homeostasis, and their activation patterns may differ between sexes due to hormonal influences or genetic factors [46]. Variations in liver function between males and females, including differences in hepatic lipid processing pathways, may also influence PFAS metabolism and its downstream effects on lipid profiles [31]. Animal studies further support these findings, with zebrafish and rodent models demonstrating that males are more susceptible to PFAS-induced disruptions in lipid metabolism, such as hepatic steatosis and dyslipidemia. For example, male rodents exposed to PFOS exhibited greater hepatic lipid accumulation compared to females, likely due to sex-specific differences in liver function and hormonal regulation of lipid pathways [46,47,48]. These findings underscore the significance of sex as a biological variable in PFAS toxicology and suggest that males may experience greater disturbances in lipid homeostasis following PFAS exposure. In contrast, human studies have not extensively examined sex-related differences in lipid metabolism following PFAS exposure. The stratified analysis conducted in this study revealed that boys had higher serum concentrations of PFAS compounds compared to girls (Table S2), which could partially explain the stronger associations observed between PFAS exposure and hypercholesterolemia risk in boys (Figure 2 and Figure 3). These findings underscore the significance of sex as a biological variable in PFAS toxicology and suggest that males may experience greater disturbances in lipid homeostasis following PFAS exposure.

This study has several notable strengths. It is one of the few to investigate PFAS exposure in adolescents, a vulnerable population group, while also exploring sex-specific differences in lipid metabolism. A key strength lies in the use of advanced statistical models such as BKMR and quantile g-computation, allowing assessment of the combined effects of multiple PFAS exposures and accounting for interactions between compounds. These models provided a robust approach to exploring non-linear relationships and complex interactions, which are often overlooked in traditional regression analyses. Furthermore, the use of data from a nationally representative sample of Korean adolescents in this study increases the generalizability of the findings.

This study had some limitations. First, the cross-sectional design restricts the ability to establish causality between PFAS exposure and lipid profile alterations; our findings primarily highlight associations rather than causal relationships. Furthermore, PFAS exposure was measured at a single time point, which may not accurately capture long-term exposure patterns. Another notable limitation is that blood samples were collected without requiring participants to fast. While we used the Martin–Hopkins formula to calculate LDL-C, which improves the reliability of lipid measurements under non-fasting conditions, fasting blood samples are still considered the gold standard for lipid analysis. The absence of fasting may have introduced variability in lipid measurements due to dietary influences or temporal fluctuations in blood lipid levels. Future studies using standardized fasting protocols are needed to improve measurement accuracy.

Additionally, while we adjusted for several confounding factors, such as age, sex, household income, and central obesity, unmeasured variables such as dietary habits, genetic predispositions, high-temperature environments, and co-exposure to other environmental pollutants were not accounted for, potentially leading to residual confounding. Although we employed advanced statistical models, such as Bayesian kernel machine regression (BKMR) and quantile g-computation, to analyze the joint effects of PFAS mixtures, our study did not explore potential synergistic interactions between PFAS and other environmental or lifestyle factors, such as dietary intake. Future longitudinal studies integrating dietary, genetic, and environmental factors are essential to validate these findings and provide a more comprehensive understanding of the complex relationships between PFAS exposure, lipid metabolism, and cardiovascular risk in adolescents.

## 5. Conclusions

In conclusion, this study suggests that PFAS mixtures play a role in lipid metabolism disruptions in adolescents, with potential sex-specific effects. Although the findings indicate an association between PFAS exposure and altered lipid profiles, further research is needed to confirm these observations and clarify the mechanisms underlying these effects. Future studies should also explore the long-term health implications of PFAS mixtures on lipid metabolism and cardiovascular risk in pediatric populations.

## Figures and Tables

**Table 1:** General characteristics of the study population.

Characteristics	Total	Boys	Girls	*p* Value
	(N = 824)	(N = 383)	(N = 441)	
Age, years	14.5 (0.2)	14.5 (0.2)	14.5 (0.2)	0.974
BMI (kg/m^2^)	22.2 (0.2)	22.7 (0.3)	21.7 (0.2)	0.001
BMI z-score	0.45 (0.06)	0.51 (0.08)	0.40 (0.08)	0.241
Waist circumference, cm	74.3 (0.6)	77.8 (0.8)	70.4 (0.6)	<0.001
Overweight/obese, %	29.7	35.2	27.7	0.49
Central obesity, %	15.8	15.1	16.5	0.661
Family income, KRW/month, %				0.57
<3000 K	19.6	18.4	20.8	
3000 K–<5000 K	37.6	37.3	37.9	
≥5000 K	42.8	44.3	41.3	
Smoking status, %				<0.001
No	93.2	89.7	96.9	
Past smoker	3.3	5.9	0.5	
Current smoker	3.5	4.4	2.6	
Alcohol consumption, ≥1 drink/month, %	7.4	8.9	5.7	0.151
Walking habits, ≥150 min/week, %	67.5	68.1	66.9	0.726
Consumption of mass-produced food items, %				<0.001
<2 times/week	24.6	18.2	31.5	
2–3 times/week	28.3	30.9	25.5	
4–6 times/week	27.8	31.3	24.1	
≥7 times/week	19.3	19.6	18.9	
Seafood consumption, %				0.734
<4 times/month	24.8	25.2	24.3	
4–7 times/month	27.7	25.9	29.7	
8–13 times/month	22.8	23.2	22.4	
≥14 times/month	24.7	25.7	23.6	
TC, mg/dL	155.8 (1.1)	150.7 (1.5)	161.2 (1.2)	<0.001
HDL-C, mg/dL	52.1 (0.4)	49.2 (0.5)	55.3 (0.6)	<0.001
LDL-C, mg/dL	83.7 (0.9)	81.2 (1.2)	105.9 (1.3)	0.001
Non-HDL-C, mg/dL	103.6 (1.0)	101.5 (1.4)	105.9 (1.3)	0.001
TG, mg/dL	96.1 (1.0)	101.0 (1.0)	90.8 (1.0)	0.007
TC ≥ 170 mg/dL, %	27.3	21.7	33.3	<0.001
LDL-C ≥ 110 mg/dL, %	8.7	6.7	10.8	0.087
Non-HDL-C ≥ 120 mg/dL, %	22.3	20.5	24.2	0.199
Hypercholesterolemia, %	29.8	24.4	35.4	0.002

Data are expressed as the mean ± SE or as percentages adjusted by survey weights. 1 K KRW is approximately 0.7 USD. Hypercholesterolemia was defined as having one of the following: TC ≥ 170 mg/dL, LDL-C ≥ 110 mg/dL, or non-HDL-C ≥ 120 mg/dL. BMI, body mass index; SE, standard error; TC, total cholesterol; LDL-C, low-density lipoprotein cholesterol; HDL-C, high-density lipoprotein cholesterol; non-HDL-C, non-high-density lipoprotein cholesterol.

**Table 2:** Combined effect of polyfluoroalkyl substance (PFAS) mixture on hypercholesterolemia using quantile g-computation models.

	OR (95% CI)	*p*-Value
Boys	1.472 (0.988, 2.191)	0.057
Girls	1.003 (0.734, 1.371)	0.985

Adjusted for age, central obesity, family income, smoking status, alcohol habits, walking habits, consumption of mass-produced food items/seafood, and menstruation years (girls). OR, odds ratio; CI, confidence interval.

## Data Availability

Access to data from the KoNEHS is restricted. Interested parties may request the data by contacting the Environmental Health Research Division at the National Institute of Environmental Research, operating under the Ministry of Environment, Republic of Korea (email: knehs@korea.kr). It is important to note that the study’s authors are not authorized to distribute the data directly.

## Supplementary Materials

The following supporting information can be downloaded at: https://www.mdpi.com/article/10.3390/toxics13020091/s1, Table S1: Distributions of PFAS concentrations (µg/L) in the study population; Table S2: Distributions of serum PFAS concentrations stratified by sex and age in the study population; Table S3: Posterior inclusion probabilities (PIPs) of serum PFAS compounds in the Bayesian kernel machine regression analysis for hypercholesterolemia risk; Figure S1: Heatmap visualizing the correlations between serum concentrations of PFAS. The Spearman’s correlation coefficient was used to quantify the associations between different PFAS concentrations, with values displayed within circles on the heatmap. PFOS, perfluorooctane sulfonic acid; PFOA, perfluorooctanoic acid; PFHxS, perfluorohexane sulfonic acid; PFNA, perfluoronanoic acid; PFDeA, perfluorodecanoic acid; Figure S2: Dose–response relationships between individual PFAS compounds and hypercholesterolemia risk using Bayesian kernel machine regression while holding all other PFAS compounds at median exposure levels; Figure S3: Relative weights of individual PFAS compounds in PFAS mixtures on hypercholesterolemia risk using quantile g computation models.
