# Supplementary material for: Concentrations of Serum Per- and Polyfluoroalkyl Substances and Lipid Health in Adolescents: A Cross-Sectional Study from the Korean National Environmental Health Survey 2018–2020
Source: Toxics. 2025 Jan 25;13(2):91. doi: 10.3390/toxics13020091 (PMC11860244; doi:10.3390/toxics13020091)
Supplement: Supplementary file 1 [file toxics-13-00091-s001.zip › toxics-3401922-supplementary.pdf]

Supplementary Materials

Table S1. Distributions of PFAS concentrations (μg/L) in the study population.

Table S2. Distributions of serum PFAS concentrations stratified by sex and age in the study population.

Table S3. Posterior Inclusion Probabilities (PIP) of serum PFAS compounds in the Bayesian Kernel Machine Regression analysis for hypercholesterolemia risk.

Figure S1. Heatmap visualizing the correlations between serum concentrations of PFAS The Spearman's correlation coefficient was used to quantify the associations between different PFAS concentrations, with values displayed within circles on the heatmap. Only statistically significant correlation coefficients ($p < 0.05$) were displayed. PFOS, perfluorooctane sulfonic acid; PFOA, perfluorooctanoic acid; PFHxS, perfluorohexane sulfonic acid; PFNA, perfluoronanoic acid; PFDeA, perfluorodecanoic acid.

Figure S2. Dose-response relationships between individual PFAS compounds and hypercholesterolemia risk using Bayesian kernel machine regression, while holding all other PFAS compounds at median exposure levels.

Figure S3. Relative weights of individual PFAS compounds in PFAS mixture on hypercholesterolemia risk using quantile g computation models.

Table S1. Distributions of PFAS concentrations (μg/L) in the study population

PFAS compounds	LOD (μg/L)	DR (%)	GM (95% CI)	Selected percentiles				
				10th	25th	50th	75th	90th
PFOS	0.056	100	7.97 (7.43 - 8.54)	4.16	5.70	7.58	10.97	15.48
PFOA	0.05	100	3.66 (3.45 - 3.88)	2.25	2.75	3.48	4.75	6.56
PFHxS	0.071	100	2.52 (2.18 - 2.91)	1.11	1.56	2.32	3.59	6.68
PFNA	0.019	100	0.92 (0.87 - 0.98)	0.54	0.70	0.92	1.20	1.67
PFDeA	0.017	100	0.45 (0.43 - 0.47)	0.29	0.36	0.45	0.56	0.72

PFAS, per- and polyfluoroalkyl substance; LOD, limit of detection; DR, detection rate; GM, geometric mean; CI, confidence intervals; PFOS, perfluorooctanesulfonic acid; PFOA, perfluorooctanoic acid; PFHxS, perfluorohexanesulfonic acid; PFNA, perfluoronanoic acid; PFDeA, perfluorodecanoic acid.

Table S2. Distribution of serum PFAS concentrations stratified by sex and age in the study population

PFAS compounds	GM (95% CI)	Percentiles	
		33rd	66th
Boys (n=383)			
PFOS	8.37 (7.71 - 9.08)*	6.40	9.69
PFOA	3.92 (3.67 - 4.18)***	3.12	4.43
PFHxS	2.84 (2.48 - 3.26)*	2.10	3.33
PFNA	0.98 (0.92 - 1.05)***	0.81	1.14
PFDeA	0.46 (0.44 - 0.49)**	0.40	0.53
Girls (n=441)			
PFOS	7.57 (7.03 - 8.15)	6.09	8.94
PFOA	3.40 (3.19 - 3.64)	2.82	3.91
PFHxS	2.22 (1.87 - 2.63)	1.55	2.68
PFNA	0.86 (0.80 - 0.92)	0.71	1.00
PFDeA	0.43 (0.41 - 0.46)	0.37	0.50

PFAS, per- and polyfluoroalkyl substance; GM, geometric mean; CI, confidence interval; PFOS, perfluorooctanesulfonic acid; PFOA, perfluorooctanoic acid; PFHxS, perfluorohexanesulfonic acid; PFNA, perfluorononanoic acid; PFDeA, perfluorodecanoic acid. The statistical significance of the differences in PFAS concentrations between boys and girls was assessed, with p-values indicated by * < 0.05, ** < 0.01, and *** < 0.001.

Table S3. Posterior Inclusion Probabilities (PIP) of serum PFAS compounds in the Bayesian Kernel Machine Regression analysis for hypercholesterolemia risk

	Male		Female	
	Group PIP	Conditional PIP	Group PIP	Conditional PIP
PFOS	0.3176	1.0000	0.3314	1.0000
PFOA	0.3610	1.0000	0.5234	1.0000
PFHxS	0.3566	1.0000	0.4512	1.0000
PFNA	0.7114	0.3551	0.6926	0.3907
PFDeA	0.7114	0.6449	0.6926	0.6093

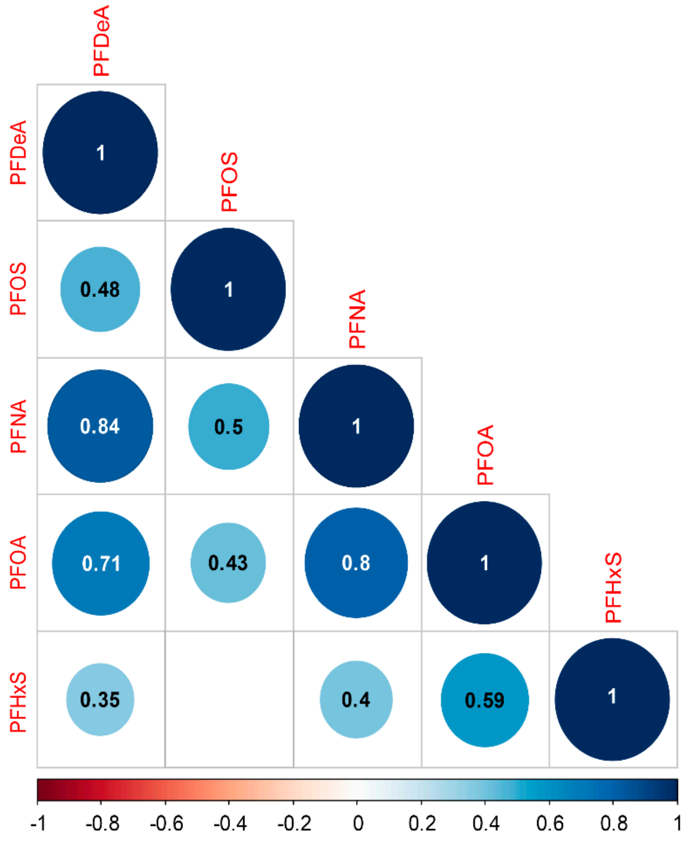

Figure S1. Heatmap visualizing the correlations between serum concentrations of PFAS The Spearman's correlation coefficient was used to quantify the associations between different PFAS concentrations, with values displayed within circles on the heatmap. Only statistically significant correlation coefficients ($p < 0.05$) were displayed. PFOS, perfluorooctane sulfonic acid; PFOA, perfluorooctanoic acid; PFHxS, perfluorohexane sulfonic acid; PFNA, perfluoronanoic acid; PFDeA, perfluorodecanoic acid.

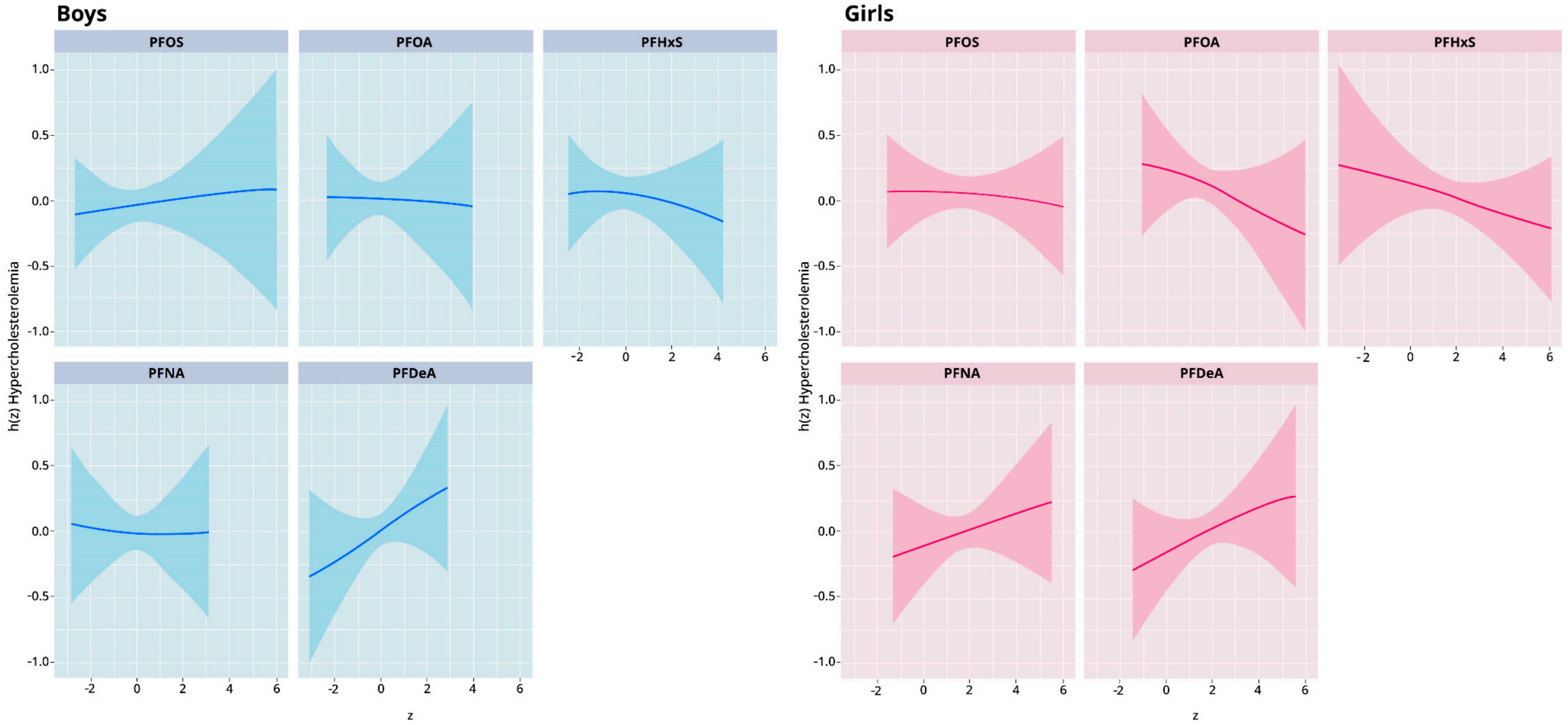

Figure S2. Dose-response relationships between individual PFAS compounds and hypercholesterolemia risk using Bayesian kernel machine regression, while holding all other PFAS compounds at median exposure levels

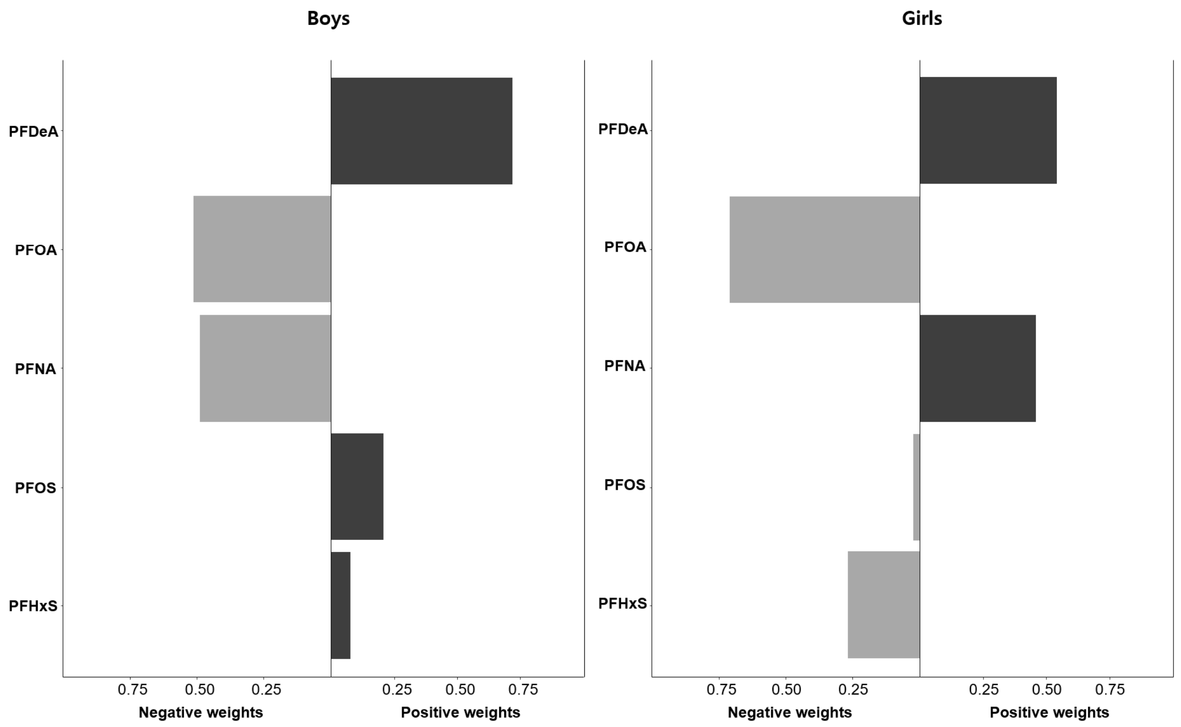

Figure S3. Relative weights of individual PFAS compounds in PFAS mixture on hypercholesterolemia risk using quantile g computation models
